# “It absolutely enlarged my world”: Exploring in-person and virtual performing arts programming for dementia care dyads

**DOI:** 10.1371/journal.pmen.0000771

**Published:** 2026-11-03

**Authors:** Ruth V. Walker, Evan G. Shelton, Cameron J. Camp, Miranda Hoffner, Katie J. Fanning

**Affiliations:** 1 Department of Psychology, The University of Tennessee at Chattanooga, Chattanooga, Tennessee, United States of America; 2 Center for Applied Research in Dementia, Lititz, Pennsylvania, United States of America; 3 Lincoln Center for the Performing Arts, New York, New York, United States of America; Instituto Federal do Maranhão: Instituto Federal de Educacao Ciencia e Tecnologia do Maranhão, BRAZIL

## Abstract

Arts-based programs are increasingly used to support people living with dementia and their care partners; however, there has been a limited focus on multi-modal, participatory performing arts programs offered in both virtual and in-person formats. We completed an evaluation of the Lincoln Center Moments (LCM) program, a free dyadic arts program grounded in four pillars: Validation/Personhood, Social Engagement, Self-Expression, and Artistic Stimulation. An exploratory mixed-methods program evaluation was conducted with 23 dyads (*N* = 46). Quantitative measures included program satisfaction, social engagement, and arts engagement. Post-session interviews were coded using an evaluative framework aligned with program pillars. Program satisfaction was high across both modalities. Care-partner-reported arts engagement for PLWD increased from pre- to post-season among the in-person subgroup but not the virtual subgroup; modality-specific analyses were exploratory. Social engagement scores were similar across formats, although in-person attendees were more likely to report friendships with others. Qualitative data supported all four LCM pillars. LCM was perceived as engaging and supportive by participants in both virtual and in-person settings. Participants who were initially hesitant to engage described greater comfort and willingness to participate over time. Participant feedback suggests that programs like LCM may provide inclusive, accessible spaces for creative engagement – particularly for those unable to attend mainstream performances. Future researchers should explore hybrid delivery formats and assess longer-term dyadic outcomes.

## Introduction

Dementia is characterized by declines in cognitive abilities that interfere with daily functioning, including social or occupational functioning [1]. These changes can also affect the psychosocial wellbeing of people living with dementia and their care partners [2]. Importantly, the wellbeing of members of the care dyad is interconnected. A systematic review of dyadic studies found that quality of life for people living with dementia and their care partners was related to individual, partner, and dyadic factors [2]. Dementia can also affect the relationship between care partners and people living with dementia. Emotional connection, shared activities and experiences, social roles, and a sense of belonging are among the factors associated with relationship quality in couples living with dementia [3]. Together, these findings point to the importance of opportunities for meaningful activity and social connection that include both members of the care dyad.

The study of arts-based interventions for people living with dementia (PLWD) and their care partners has expanded significantly in recent years. Across modalities (e.g., visual arts, music, dance, drama, storytelling), studies suggest arts engagement can support mental health [4–6], quality of life [7–9], social connection [6,8–10], cognitive functioning [4], and reductions in responsive behaviors [11], among PLWD. Quinn and colleagues’ systematic review found improvements in depression and anxiety for PLWD across a variety of modalities, including dance, music, and visual arts [5].

Arts programming for PLWD increasingly includes cultural dementia care, or arts-and-culture programming offered through community cultural institutions. This approach positions arts participation as an accessible, relationship-centered support rather than solely a clinical intervention [12]. A systematic review of arts and culture-based interventions reported improvements in well-being outcomes for PLWD [13]. Historically, however, cultural programming has been limited to in-person formats, which can create barriers to participation such as transportation and mobility, stigma, and care-related demands [14]. In the current study, we evaluated a performing arts program designed for PLWD and their care partners, with both in-person and virtual modality opportunities. We examined participant experiences related to validation/personhood, social engagement, self-expression, and artistic stimulation.

### Dyadic and participatory arts programs

There has been a growing recognition of the need for dyadic interventions that consider the interconnected wellbeing of PLWD and their care partners. As dementia progresses, care partners often experience parallel emotional and psychological challenges [11]. A systematic review and meta-analysis of dyadic sensory art therapies (e.g., music therapy, storytelling, art therapy, dance therapy) for PLWD found significant improvements in responsive behaviors, caregiver burden, and relationship quality; however, they did not find support for improvements in depression or quality of life for care partners or PLWD [11]. Anxiety was only measured in PLWD, and the findings were mixed. These quantitative findings suggest promising effects in some domains but also highlight limitations in current measurement approaches and outcomes.

Researchers have found it challenging to assess dyadic outcomes using quantitative methods alone. Challenges include increased burden on care partners, uncertainty about respondent perspective, and small sample sizes due to recruitment and retention difficulties [8]. Alternatively, qualitative evaluations such as the study of the *B-Sharp Music Wellness* help us understand participant experiences that are not captured by standardized scales. PLWD and their care partners in that study reported a positive impact on their quality of life, positivity, program engagement, relationship with their care partner, and social connections within their community. Participants highlighted how the program normalized participation in public cultural events, reducing feelings of isolation and stigma commonly associated with dementia.

Similarly, McManus et al. conducted a mixed methods evaluation of an eight-week multimodal performing arts intervention for caregivers of PLWD that combined music, theater, and dance activities [15]. The program provided opportunities for dyads to co-create and participate in the arts. Although researchers found a significant reduction in caregiver burden quantitatively, there were no changes in caregiver resiliency or quality of life for both care partners and PLWD. However, in their qualitative analyses they found support for the appreciation of the normalization of structured social activities, opportunities for the PLWD to interact without dependency, and a chance for care partners to see their loved one engaged in activities and discussions that reminded them of the PLWD’s capabilities.

### The Lincoln Center Moments program

The Lincoln Center Moments (LCM) program is designed to foster relational wellbeing through shared aesthetic experiences grounded in four core pillars: Validation/Personhood, Social Engagement, Self-Expression, and Artistic Stimulation. Validation/Personhood emphasizes recognizing each participant as an individual and honoring their identity and lived experiences. Social Engagement focuses on creating opportunities for participants to connect with one another and experience a sense of community. Self-Expression emphasizes autonomy and opportunities for participants to make choices and contribute something of themselves to the experience. Artistic Stimulation encourages creativity, learning, and artistic exploration, with an emphasis on imagination rather than memory. The LCM approach to arts-based engagement prioritizes shared experiences for both PLWD and their care partners, rather than providing separate or respite-focused activities. LCM features renowned artists from Lincoln Center’s resident organizations and beyond, intentionally avoiding simplified or separate programming to welcome both members of the dyad as equal participants in world-class music, dance, and theater events. In both in-person and virtual sessions, audiences are given opportunities to interact with the performers and ask questions.

Across the Fall 2023 and Spring 2024 seasons under evaluation, LCM offered eight programs per season, with four delivered in person and four virtually. Programs were offered approximately every two to three weeks. In-person performances included original musical theater compositions in English and Spanish with Florencia Cuenca and Jaime Lozano; small ensemble classical performances with the New York Philharmonic; Hispanic/Latine dance with Ballet Hispánico; protest songs of the 1960s performed by singer-songwriter Morley; salsa music and dancing with all-female Lulada Club; a tribute to Nat King Cole with Jazz at Lincoln Center; contemporary bluegrass string quartet compositions and improvisation with Invoke (who also presented a program virtually); and classical music celebrating spring with the Calidore String Quartet and the Chamber Music Society of Lincoln Center.

Virtual performances included a recorded concert with Jazz at Lincoln Center celebrating the life and experience of bassist Bill Crow; a contemporary re-interpretation of Dizzy Gillespie’s *Night in Tunisia* with Yacine Boularès; a meditation on cellist and vocalist Leyla McCalla’s professional journey; a screening of American Ballet Theatre Studio Company’s recorded work *The Go Between;* operatic characterization in *Roméo et Juliette* with the Metropolitan Opera; a playful exploration of music to relay sensory experiences with the Juilliard School’s Dolphins Quartet; and a preview of a musical work-in-progress by Alphonso Horne telling the story of charismatic Ghanaian-American leader Mother Kofi.

In-person programs were held in the Stanley H. Kaplan Penthouse at Lincoln Center. Two approximately one-hour performances were offered for each program. The 11:00 a.m. performance was a relaxed performance designed for a broader neurodiverse audience, including people impacted by dementia and adults with developmental disabilities. The 1:00 p.m. performance was followed by an approximately one-hour interactive workshop specifically designed for audiences impacted by dementia. Interactive workshops were collaboratively planned and implemented by a team of facilitators with backgrounds in music therapy and teaching artistry across disciplines, including dance, music, visual art, and theater/storytelling. Facilitators had professional experience working with people living with dementia through clinical music therapy practice, museum or performing arts programming, and other settings. They also regularly participated in professional development with expert practitioners in this area. Workshop planning emphasized accessibility, with facilitators preparing multiple ways for participants to engage in activities based on their communication and physical abilities. In-person workshops were conducted in multiple rooms to maintain smaller groups, with approximately two to four members of the facilitation team in each room to support implementation.

Workshop activities regularly incorporated music and art exploration and welcomed participation across levels of ability, including verbal and nonverbal participation and participation by individuals with varying levels of mobility. Activities encouraged collaborative expression and co-creation, providing opportunities for mutual engagement and self-expression. For example, during in-person workshops during the seasons under investigation, participants collaboratively built a soundscape inspired by a spring rainstorm, using handheld instruments and their bodies, felt the connection between each other through a guided movement activity using an octaband, and contributed individually written/designed postcards to a time capsule to send a message to a loved one in the future. Consistency across workshops was supported through collaborative planning, shared program pillars, facilitator training and professional development, preparation of materials and activities, and co-facilitation. However, the program intentionally allowed facilitators to adapt activities and modes of participation to participants’ communication, cognitive, and physical abilities.

Virtual programs were approximately 90 minutes in duration and integrated the artistic performance and facilitated workshop activities within a shared Zoom meeting. This format allowed for greater structural variety. During the seasons under investigation, participants began a program by sharing an object in their home that is meaningful for them to begin the exploration of storytelling with bluegrass quartet Invoke; participants contributed feelings-based words for musician Leyla McCalla to improvise a new song reflective of the room; and participants created full landscape images beginning only with dots and lines while listening to live music. Already in their 10^th^ year, this program has addressed the call for sustained, ongoing participation—rather than one-time or short-term interventions—to help participants maintain benefits over time [5,15]. Finally, the delivery in both in-person and virtual formats increases accessibility, ensuring dyads can participate regardless of location or mobility status. It is also entirely free.

### Theoretical framework

Relational wellbeing refers to a sense of mutual connection, trust, and engagement that may develop from meaningful social relationships, particularly between informal care partners and PLWD [16,17]. Although person-centered care emphasizes individualized needs and dignity (often focusing on the person with dementia alone), relational-centered approaches shift the focus to shared experiences and the reciprocal nature of care relationships (16). This theoretical framing highlights how shared activities can support reciprocity, joint attention, mutual recognition, and continuity of the relationship narrative despite changing interpersonal roles [18]. Hatton urges arts-based researchers and artists to consider, “The role of the arts in supporting different types of relationality” [16], and emphasizes the importance of attending to the social, environmental, and contextual factors that shape the everyday lives of people living with dementia. In the context of the Lincoln Center Moments program, shared aesthetic experiences may provide opportunities for connection and mutual understanding within dyads, not just individual engagement.

### The current study

Despite promising findings from arts-based work with PLWD, there is still limited research on participatory performing arts programs compared to visual arts and music therapies [4,7,19]; further, participatory performing arts programs, particularly those focused on dyads, are underrepresented [8,15]. As virtual programming becomes more prevalent in a post-COVID world, there is also a growing need for evaluations comparing virtual and in-person formats. Our goal with the current study is to respond to the call for more mixed methods research on arts-based interventions [20], particularly given the conflicting findings sometimes described by researchers comparing results on quantitative measures and qualitative interviews when evaluating arts-based programs [8,9]. Specifically, we evaluated LCM using quantitative measures of program satisfaction, engagement with the arts, and social engagement alongside qualitative interviews with PLWD to explore how participant experiences aligned with our program pillars for both in-person and virtual programming.

## Methods

### Ethics statement

This study was approved by the independent Institutional Review Board of Linda and Cameron, Inc. (IRB00012209) on September 6, 2023. All participants provided written informed consent prior to participating in the study.

### Study design

For this study, we used an exploratory mixed-methods design to evaluate participant experiences and outcomes associated with LCM among dyads consisting of PLWD and their care partners. Data were collected from participants during the Fall 2023 and Spring 2024 seasons through post-workshop surveys and brief interviews. Please see our Open Science Framework Registration for additional information regarding the program and study design [21].

### Participants

Participants were recruited from LCM attendees during the Fall 2023 and Spring 2024 seasons. Recruitment procedures differed somewhat between seasons. Fall 2023 served as the initial pilot. Recruitment included an interest box with program registration, flyers, and email solicitation beginning September 15, 2023. Six dyads participated during the Fall season. Recruitment and enrollment were tracked more systematically during Spring 2024.

For Spring 2024, an optional question was included on the LCM registration form that allowed attendees to indicate interest in the research study. A total of 72 dyads expressed initial interest. After applying eligibility criteria based on registration for a post-performance workshop and registration for at least two programs during the season, 48 dyads were eligible and contacted. The research team used an initial email, at least two follow-up telephone calls, a second email, and a final telephone call if needed. Three information sessions were also offered, including two in-person sessions and one virtual session. Reasons for non-participation among eligible dyads were not systematically collected.

Recruitment procedures were modified for Spring based on experiences during the Fall pilot. The minimum attendance requirement was reduced from all four programs to at least two programs. Participants also received compensation for completing research activities. Seventeen dyads participated during Spring 2024, including 11 in-person and six virtual dyads. Two additional Spring dyads completed baseline assessments but did not attend LCM programming or complete follow-up assessments and therefore were not included in the final sample. Across both seasons, the final sample included 23 dyads (*N* = 46 individuals), with 15 participating in in-person programming and eight participating virtually. Fig 1 summarizes participant recruitment, eligibility, participation, and retention across the Fall 2023 and Spring 2024 seasons. Across cohorts, PLWD ranged in age from 67-94 (*M* = 81.10, *SD* = 8.97), and care partners ranged from 33-86 (*M* = 65.96, *SD* = 14.02). See Table 1 for full demographic details.

**Table 1:** Participant demographics.

		Fall 2023 (dyads = 6)		Spring 2024 (dyads = 17)	
**Factor**		*Participants*	*Care Partners*	*Participants*	*Care Partners*
**Modality**					
	In-Person	4	4	11	11
	Virtual	2	2	6	6
**Gender**					
	Man	2	1	7	2
	Woman	4	5	9	13
**Race/Ethnicity**					
	White Non-Hispanic	3	4	14	13
	White Hispanic	0	1	0	0
	Black/African-American	1	1	2	3
	Other	1	0	0	0
**Dyad Relationship**					
	Spouse	2		9	
	Professional Care Partner	2		1	
	Parent/Adult Child	2		4	
	Sibling	0		1	
	Friend	0		1	
**Education**					
	Less than High School	0	0	1	1
	Some High School	1	0	0	0
	High School Graduate	0	0	1	0
	Some College	0	2	1	1
	College Graduate	2	2	4	7
	Post-College Graduate	2	4	9	7

Note. Counts may not sum to cohort totals due to missing demographic data.

**Fig 1:**
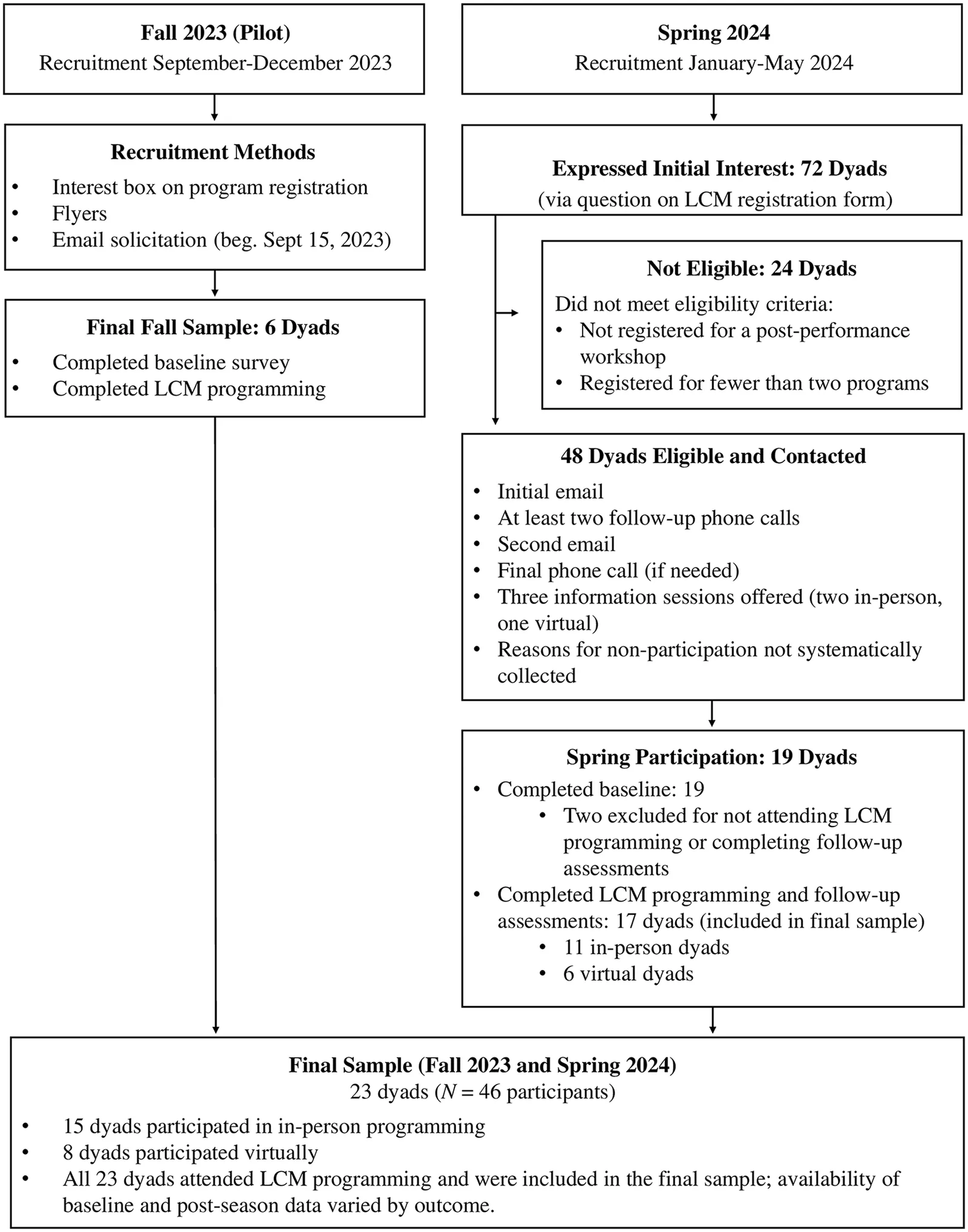
Participant recruitment and inclusion across the Fall 2023 and Spring 2024 cohorts.

We did not assess participants’ type or severity of dementia, nor did we require clinical documentation of diagnosis. The goal of the program evaluation was to understand participant experiences of arts-based workshops offered through Lincoln Center Moments (LCM), which are broadly marketed to individuals living with dementia and their care partners. As such, attendance and participation were not restricted based on diagnosis or impairment level, and all interested individuals who self-identified or were identified by a care partner as living with memory loss were eligible to participate.

### Procedure

All participants enrolled in the study completed written informed consent. Care partners completed a demographic questionnaire and baseline and final surveys administered before the first program and following the end of the season, respectively. These surveys included items assessing care partners’ own experiences as well as items asking them to report their perceptions of the PLWD’s engagement and well-being. Thus, items completed by care partners about the PLWD reflect the care partner’s perspective rather than direct self-report by the PLWD.

PLWD completed brief interviews immediately following each program they attended. These interviews included open-ended questions about their experiences as well as brief quantitative rating items assessing program satisfaction and social engagement. Interviews were conducted by a Lincoln Center staff member hired specifically to oversee research activities for this study, including recruitment, interviewing, transcription, and participant follow-up. This staff member was not involved in the development, facilitation, or implementation of LCM programming. The interviewer was present with the PLWD during the interview. Care partners were not included in the post-program interviews. Although pre-post questionnaires are commonly used to assess changes in well-being among PLWD, we intentionally limited longitudinal assessments to care partners to reduce cognitive burden on PLWD and concerns regarding retrospective assessment over longer time frames. Instead, PLWD provided feedback immediately following each program, prioritizing accessibility and reflection grounded in the present moment.

The Lincoln Center staff member hired to manage study activities conducted the interviews, prepared interview transcripts, and removed identifying information before providing qualitative data to the broader research team for analysis. Survey data were collected electronically, and any identifying information was also removed by Lincoln Center staff before the data were provided to the research team. The research team responsible for developing the study measures and conducting all analyses was independent of Lincoln Center and was not involved in LCM program development or implementation.

Likert-scale questions used a 4-point response scale (0 = *strongly disagree* to 3 = *strongly agree*). This approach intentionally removed a neutral midpoint, encouraging participants to reflect on their experiences rather than default to a neutral option. This was considered appropriate for the target population, as individuals living with dementia may be more likely to select neutral options or respond in socially desirable ways without reflective processing [22]. Visual choice cards were used as needed to assist PLWD with comprehension and accessibility of response options. Baseline and final surveys, as well as post-workshop interview guides, are available in the project’s OSF repository.

### Measures

The quantitative measures used in this program evaluation were developed specifically to assess participants’ experiences in relation to the four LCM program pillars. Item development was informed by focus groups conducted during Summer 2023 with PLWD, care partners, and LCM program facilitators. Feedback from these groups was used to identify experiences and outcomes relevant to the program’s intended goals and to develop items that were brief and accessible for the study population. Because the primary purpose of the study was to evaluate whether LCM was meeting its intended program goals, study-specific measures were selected rather than broader standardized measures that did not directly map onto the four program pillars. Internal consistency estimates for the multi-item measures are reported below.

**Program Satisfaction.** Program satisfaction was assessed using three 4-point Likert scale questions that ranged from 0 (*strongly disagree*) to 3 (*strongly agree*). A sample item included, “The performance captured and held my interest.” PLWD rated program satisfaction after each program and ratings were averaged across all programs attended. A Cronbach’s alpha of .78 was obtained for these items.

**Engagement with the Arts.** Arts engagement was assessed using eight 4-point Likert scale questions that ranged from 0 (*strongly disagree*) to 3 (*strongly agree*). A sample item included, “The arts are currently an important part of my life.” Engagement with the arts was assessed at baseline and post-season. Care partners completed the measure both with respect to their own arts engagement and as a proxy report of the PLWD’s arts engagement. Scores were averaged across all eight items. A Cronbach’s alpha of .86 was obtained for the current study.

**Validation/Personhood.** Validation and personhood were assessed using an open-ended question during the post-performance interviews. PLWD were asked questions such as, “Reflecting on your interactions with everyone at Lincoln Center today, how did they contribute to creating an environment for you to learn and experience new things?”

**Social Engagement.** Social engagement or inclusion was assessed using five 4-point Likert scale questions that ranged from 0 (*strongly disagree*) to 3 (*strongly agree*). Sample items included, “I have friends here.” PLWD rated social engagement after each program and ratings were averaged across all programs attended. A Cronbach’s alpha level of  .73 was obtained with the current sample. Additionally, PLWD were asked, “Can you tell me how the program helped you connect with others today?” during the brief, post-performance interviews.

**Self-Expression.** Self-expression was assessed using an open-ended question during the post-performance interviews. PLWD were asked, “Has today’s program influenced your confidence or desire to participate in more artistic activities?”

**Artistic Stimulation.** Artistic stimulation was assessed using open-ended questions during the brief, post-performance interviews. PLWD were asked, “Did today’s program influence your appreciation for new forms of music, art, or performance?” and “Thinking about today’s program, can you share something you learned today?”

### Data analysis

Evaluative coding, applied deductively, was used to assess whether participants perceived the LCM program was fulfilling the program pillars: Validation/Personhood, Social Engagement, Self-Expression, and Artistic Stimulation. Evaluative coding is particularly useful in program evaluations because it allows for a structured assessment of a program by focusing on predefined goals (i.e., program pillars) and using participant feedback to assess the extent to which those goals were met [23]. Deductive coding methods such as evaluative coding can help identify how well a program meets its intended objectives by linking participant feedback to clearly defined evaluative criteria (i.e., program pillars) [24].

Two researchers with experience in qualitative research conducted the coding. The coders collaboratively coded the Fall 2023 data to develop and refine an initial codebook based on the four pillar definitions and establish a shared understanding of how the evaluative criteria should be applied. Formal inter-rater reliability was not calculated because coding was conducted using a collaborative, consensus-based approach rather than independent coding intended to estimate statistical agreement. Coding decisions and areas of uncertainty were discussed and refined collaboratively to promote consistency across coders. Once the codebook and coding approach were established, the full dataset was coded for alignment with the program pillars and for additional patterns we identified inductively from participant responses.

No qualitative analysis software was used. Microsoft Excel was used to organize codes by program pillar and link codes with supporting participant quotations. The complete codebook is publicly available through our Open Science Framework (OSF) repository and includes participant-level quotations associated with each code, allowing readers to examine the textual evidence underlying each of our coding decisions. This provides a transparent audit trail of how participant responses were interpreted and categorized within the evaluative framework, supporting transparency and transferability.

Quantitative analyses were conducted using JASP. Planned pre-post comparisons were conducted for quantitative outcomes assessed longitudinally. Further independent sample comparisons were conducted to explore differences between participants attending virtual and in-person programming. Participants were not randomly assigned to modality; therefore, analyses describe differences between modality groups rather than estimate the effect of delivery modality. Multi-item scale scores were calculated by averaging responses across items. Although individual items used 4-point ordinal response scales, analyses of multi-item measures were conducted using composite scores. Given the small sample size, quantitative analyses should be considered exploratory and are interpreted cautiously alongside effect sizes, confidence intervals, and qualitative findings. Statistical findings are interpreted as preliminary rather than as evidence of program effectiveness. Given the small analytic samples, nonparametric sensitivity analyses were also conducted to assess whether conclusions were robust to methods requiring fewer distributional assumptions. Wilcoxon signed-rank tests were used for paired pre-post comparisons, and Mann-Whitney U tests were used for between-group comparisons.

No missing values were imputed. Analyses were conducted using available observed data, and participants were included in each analysis when they had the data required for that analysis. Thus, analytic sample sizes vary across tests. For paired samples analyses, only participants with both baseline and post-season data for the relevant outcome were included. Analytic sample sizes are reported with each analysis in the Results. Quantitative data and analyses are available on our OSF repository.

## Results

### Overall program evaluation

Program satisfaction was high across both modalities. Virtual participants reported descriptively higher satisfaction than in-person participants, but exploratory between-group comparisons were not statistically significant (see Table 2). Program attendance and ratings varied across performances and modalities. The highest-attended in-person event was Spring 2024 Performance 3 (Music of Nat King Cole), with nine of 17 in-person dyads (52.9%) attending and completing post-performance surveys and interviews. The highest-attended virtual event was Spring 2024 Virtual Performance 2 (*Roméo et Juliette* with the Metropolitan Opera), with five of six virtual dyads (83.3%) attending and completing surveys.

**Table 2:** Independent and Paired Samples t-Test Results by Modality.

Outcome Measure	Modality	*n*	Pre (M, SD)	Post (M, SD)	*t(df)*	*p*	Cohen’s *d* [95% CI]	Mean Difference [95% CI]
Arts Engagement (PLWD, Care-Partner Report)	In-Person	13	2.27 (.61)	2.54 (0.54)	-2.81(12)	**.016**	-0.78 [-1.39, -.14]	-.27 [-.48, -.06]
	Virtual	7	1.93 (0.85)	2.07 (.47)	-0.68(6)	.522	-0.26 [-1.00, .51]	-.14 [-.66, .37]
Arts Engagement (Care Partner Self-Report)	In-Person	13	2.41 (.52)	2.54 (0.58)	-0.92(12)	.375	-0.26 [-.80, .30]	-.13 [-.43, .18]
	Virtual	7	2.18 (0.66)	2.32 (0.51)	-0.44(6)	.673	-0.17 [-.91, .59]	-.14 [-.93, .65]
Program Satisfaction	In-Person	14	–	2.48 (0.24)	-1.49†(19)	.152	-0.69 [-1.62, 0.25]	-.17 [-.40, .07]
	Virtual	7	–	2.64 (0.24)				
Social Engagement	In-Person	14	–	2.37 (0.26)	0.17†(19)	.864	0.08 [-.83, .99]	0.02 [-.22, .26]
	Virtual	7	–	2.35 (0.22)				

**Note.** PLWD = Person Living with Dementia. *t* values for pre-post comparisons reflect paired samples t-tests. †Independent samples t-test comparing in-person and virtual groups on post-test scores. Cohen’s *d* values reflect within-group effect sizes for paired comparisons and between-group effect sizes for independent comparisons.

The highest program satisfaction ratings were observed for Spring 2024 Virtual Performance 4 (Artists at the Atrium: Mother Kofi; *M* = 3.00, *SD* = 0.00) and Fall 2023 In-Person Performance 3 (Ballet Hispánico; *M* = 3.00, *SD* = 0.00). Note that these are perfect scores, meaning all participants responded “strongly agree” across all program satisfaction items. In contrast, the lowest satisfaction was reported for Fall 2023 Virtual Performance 4 (Leyla McCalla; *M* = 2.00, *SD* = 0.00). This score still reflects a positive response, with all participants selecting “agree.”

### Validation and personhood

A total of three virtual participants (43%) and 10 in-person participants (71%) across the Fall 2023 and Spring 2024 seasons described how LCM staff recognize and value them as individuals. Participants described how workshop facilitators demonstrated this by first welcoming and greeting each person. For example, an in-person participant said, “Everybody was welcoming, and I felt very comfortable in this environment...It just seemed to include anybody, like there were no boundaries” (92-year-old woman). This participant highlighted that there were no boundaries to make them feel excluded and that they were welcomed by “everybody,” which implies all LCM staff and not just workshop facilitators. This was reiterated by another participant who noted, “The facilitators, all of the people who are involved with the program are great. Friendly. Eye contact. Helpful. Super helpful” (81-year-old woman). This welcome went beyond just greeting and acknowledging participants. One in-person attendee explained how staff preparations for their return to the program made them feel a sense of belonging.

It’s funny because they made me feel like I was one of the oldsters in the group because when I came in, they had my paperwork separate because it was like, already almost done already because they knew I was coming. So, it made me feel like I was part of the, you know, part of the whole thing, which was kind of good. It feels nice (70-year-old man).

Participants also highlighted how LCM staff gave everyone an opportunity to share and made the program accessible (e.g., virtual program, multiple ways to participate). As one participant explained, “I thought facilitators do a nice job of asking questions, thought provoking questions. And the more other participants willingly respond or share their ideas. It makes me more willing to consider doing that too” (71-year-old man, Virtual Attendee). This participant pointed out that the facilitators not only encourage sharing thoughts and ideas, but also that as a participant they feel more comfortable with the possibility of contributing when they see other PLWD engaging. Another in-person participant described how accepting the program facilitators are, “Everybody was very accepting. They accepted anything anybody had to say. And I mean, there were some people there that were a little bit not into it as much as others and even those were accepting” (70-year-old man). This quote illustrates how facilitators are working to meet participants where they are, accepting participants’ varying levels of comfort while engaging in the workshop discussions and activities, even those who were “not into it.”

Finally, one participant attempted to describe how attending the virtual programs impacted their feelings of self-worth and connectedness in the following quote.

I’m immune suppressed, since COVID hit. I have spent the greater portion of my life sequestered in my apartment, or my daughter’s apartment. The people that I mainly interact with are my two daughters. Being reminded of the broader world of all the other people out there who may be dealing with immune suppression, or Lewy body dementia, or other things that I’m experiencing makes me feel more whole. Whereas I felt since COVID, I felt diminished, and then Lewy body dementia came along. And if anything’s going to make you feel dementia diminished, that is it. To have your brain start playing tricks on you is a very humbling, diminishing thing. And so, seeing again, all these other people who perhaps are living with many of the same challenges I live with, I don’t even know. I’m a writer, but there’s not words to express exactly how nourishing and affirming that is for me (67-year-old woman, Virtual Attendee).

For this participant, attending the program allowed them the opportunity to connect virtually with other people that are experiencing similar challenges, an experience that felt affirming and validating to their sense of self.

### Social engagement

Average social engagement scores were similar among virtual and in-person participants, with no statistically significant difference between groups (see Table 2). In exploratory item-level analyses, we found in-person participants (*n* = 14) reported higher scores on the item “I have friends here” (*M* = 2.24, *SD* = 0.33) compared to virtual participants (*n* = 7; *M* = 1.58, *SD* = 0.74), *t*(19) = 2.83, *p* = .011, *d* = 1.31, 95% CI [0.30, 2.29]. A Mann-Whitney U sensitivity analysis supported the same conclusion (*U* = 81.50, *p* = .011). No statistically significant group differences were observed for the remaining social engagement items (all *p*s > .05).

In post-performance interviews, most virtual participants (*n* = 6, 86%) and in-person participants (*n* = 11, 79%) described how the LCM program helped them connect and engage with others. Through shared experiences, participants connected with their care partners, fellow attendees, performers during Q&A sessions, and Lincoln Center staff. Participants particularly valued the communal aspects of the program, such as the shared audience experience during performances and workshops where they could exchange stories, ideas, and creative expressions, making them feel part of a supportive and inclusive environment. For example, one woman stated, “Going to the art room, you know, we go in as strangers, but we get in together and talking and exchanging ideas. You all become friends” (woman, In-Person Attendee, no age provided). For some, friendships have developed. For others, “A sense of belonging,” (82-year-old man, In-Person Attendee) and camaraderie, “I like the camaraderie. Being together and sharing ideas, I like that” (70-year-old woman, In-Person Attendee).

One participant even described becoming a source of guidance for others, noting, “There were people there around me also that were new, or looked like they were fairly new to the program, and they were asking questions, and I was kind of answering them. So I became...A source” (70-year-old man, In-Person Attendee). Another participant emphasized how different people enjoying different types of music helped build comfort, stating, “It’s a different audience and a different kind of feeling that I get when looking around and seeing how different people enjoy different types of music, and it helps me to reinforce my comfort level” (87-year-old woman, In-Person Attendee). These reflections are closely tied to the pillar of personhood and validation. For one participant (70-year-old man), becoming a source of guidance for others not only allowed him to contribute to the community but also reinforced his sense of identity and worth, validating his ability to help and support others. Similarly, another participant’s (87-year-old woman) feelings of comfort in observing the diverse experiences of others reflect a validation of her own preferences and emotional responses, not only fostering a sense of belonging but also reinforcing her personhood.

Although the modality and experiences are different, virtual attendees also described how the program helps them connect with others. As one 67-year-old woman explained, joining the virtual program, “Absolutely enlarged my world, which sometimes feels far too small...” She went on to note that she attends the program with her daughter. “It’s been wonderful because I love to hear her ideas, and to view her creativity, and to learn from her experience… And it’s wonderful to connect with her in that way.” She also described how the program helped her feel connected with the other program participants.

I don’t get the opportunity, very often, to be around children anymore. And I miss that. I miss that so much. So, it’s wonderful to see other faces that look about my age, and to hear the things coming from people who are my age, but it’s especially wonderful when I hear their caregivers, or a granddaughter. And you know, I’m reminded that there are other people in the world, and they have different opinions and ideas and that’s wonderful to me. I love that.

She not only appreciated the opportunity LCM provides her to connect and see people her own age, but also the chance to connect with young people that attend with the other program participants visible on Zoom.

Participants also described how social engagement and connection is facilitated through music, art, and creativity. As one woman said, “When we can connect on those levels, it helps us to bridge things that might otherwise divide us” (67-year-old woman, Virtual Attendee). She went on to recount how the program has provided opportunities for her to experience deeper emotional connections with her family.

My other daughter lives down the hall from us. And she came in during the program and it is so powerful and so moving that it moved her to tears. Feeling that family connectivity, that emotional energy, I’m just going to be...that’s going to carry me for the next to however long, I guess until next fall when you start again. It’ll have to carry me because I’m going to miss you guys so much.

When asked how the program helped them connect with others, some participants felt they had opportunities to connect with their care partner, but not other attendees. For example, a 92-year-old woman who attended in person stated, “I was with my caretaker, and I paid more attention to what she was saying and doing.” This was reiterated by another participant, “Well, I just came with my sister so if I go alone, most likely, I seem to connect with other people. But I’m with her. So, you know, we’re talking together” (70-year-old woman, In-Person Attendee). One participant acknowledged that she struggles to connect with others in general, saying “I’m slow to connect for sure” (81-year-old woman, In-Person Attendee). These participants highlight that although they may not have connected with others attending the program, they still had opportunities to connect with their care partner.

Considering both the quantitative and qualitative findings adds context to how participants experienced social connection across formats. Overall social engagement scores were similar, suggesting that PLWD in both modalities generally felt socially engaged within LCM. However, in-person participants reported higher scores on the specific item “I have friends here.” This pattern was also reflected in the open-ended responses. In-person participants more often described developing friendships, connecting with other attendees, and becoming part of a group. Virtual participants described connection through existing relationships, supporting others, and feeling connected to a broader community. Together, this may suggest that social connection was present in both formats but that it took somewhat different forms.

### Self-expression

The majority of PLWD participating in-person (*n* = 12, 86%) and virtually (*n* = 6, 86%) described in their interviews how LCM provided them with opportunities for self-expression through workshop activities and discussion. Participants described the role the facilitators played in creating an environment conducive to self-expression by offering participants unconditional positive support, encouraging them to explore their creativity without fear of judgment. For example, as quoted in the validation pillar previously, “They accepted anything anybody had to say” (70-year-old man). This supportive atmosphere not only enhanced their artistic interests but also bolstered their confidence and sense of identity. By validating participants’ creative expressions, the program reinforced their personhood, helping them feel seen, heard, respected, and valued. Thus, participant responses illustrate how the encouragement of self-expression connects to feelings of validation and personhood, showing how both pillars were intertwined in participants’ experiences of the program.

Participants also highlighted the emotional significance of these opportunities for self-expression. One virtual attendee, reflecting on the program’s focus on artistic interpretation, noted, “Everybody sees different things in the artwork... It allows me to reflect and say, ‘oh, okay, yeah I saw that too, but I didn’t think of it in that term’” (71-year-old man). The same participant described the process of making creative connections across modalities, stating, “I’m learning how to connect music and art. And how the mood of the music can influence the art project.” These reflections illustrate how facilitators invited participants to engage in deeper interpretation and reflection, connecting their personal insights with multiple artistic expressions and fostering a shared sense of understanding.

Some participants described how the program helped them rediscover creative parts of themselves. As one virtual attendee shared, “It’s reminded me of the things that I love the most about myself, and that is my creative instincts... it also reminded me that it’s okay for me to attempt creative things even though I’m terrible at them” (67-year-old woman). She added that, “It was fun to try without judging myself as being a terrible visual artist.” This highlights the role of the program in creating a nonjudgemental space that encouraged creative risk-taking, helping participants reconnect with aspects of their identity that may have been dormant. This same participant described in a subsequent interview how this freedom to explore without judgment helped them overcome self-imposed limits, “It has encouraged me not to think in limits... to think outside of the limits I’ve always imposed on myself.” This highlights how self-expression in the LCM program helped expand her sense of what is possible.

Participants also described moments of creative motivation, both through rediscovery and new discovery. One in-person attendee shared, “I feel inspired to pick up my flute today... I’m going to pick up my flute when I go home” (70-year-old woman). This suggests a re-engagement with a former passion, even if the modality (music performance) differed from the program’s focus. Another participant described the discovery of artistic confidence, stating, “I was wary of the workshop activity and shy about them and I am less so now. I’ve always wanted to draw and made me think maybe I should take that up” (81-year-old woman, in-person attendee). This illustrates how the program supported both those returning to past creative outlets and those discovering new ones, fostering self-expression regardless of prior artistic experience and inspiring continued artistic engagement beyond the program setting.

### Artistic stimulation

PLWD arts engagement reported by care partners increased from pre-to-post season (see Table 2). We conducted exploratory analyses by modality and found the increase present for in-person participants but not PLWD who participated virtually. Self-reported arts engagement for care partners did not significantly change. Wilcoxon signed-rank sensitivity analyses produced the same pattern of results, with a significant in-person increase in care-partner-reported PLWD arts engagement (*p* = .022); other pre-post comparisons were not statistically significant.

All of the in-person participants (*n* = 14, 100%) and virtual participants (*n* = 7, 100%) across the Fall 2023 and Spring 2024 seasons described how LCM encouraged them to learn, experience, explore, and appreciate music, art, and performance during their post-program interviews. Artistic stimulation sparked curiosity, joy, and engagement with both familiar and new art forms. As one virtual participant shared, “I never heard music like that before, and I enjoyed it” (93-year-old woman). Another participant echoed this sentiment, saying, “I hadn’t seen a lot of this type of art before and I really enjoyed it” (71-year-old man, Virtual Attendee), reflecting the program’s role in expanding their exposure to diverse art forms.

The opportunity to engage and broaden their artistic horizons was meaningful for participants. As one in-person participant shared, “Lincoln Center - the arts mean something to me. Enjoyment. I liked the jazz... I like to dress up and go to the building” (70-year-old woman). This participant went on to highlight how the program is meaningful not only because it fosters enjoyment by giving them a reason to dress up and leave their home, but also because it supports their intellectual growth through artistic stimulation, “It helps me to enrich…and it helped my knowledge.” Another in-person attendee highlighted how accessible the program is, “This kind of music and dance appreciation can be enjoyed by anybody. You don’t need training” (92-year-old woman). Another participant observed how the performances and follow-up workshops are helping them see the connections between different forms of arts, saying, “I’m learning how to connect music and art. And how the mood of the music can influence the art project” (71-year-old man, Virtual Attendee).

Some participants stated the diverse programs offered by LCM inspired them to explore new art forms. As one virtual participant described, “It’s like every time we do these programs, it seems like something new is being brought up… so for me as it increases my awareness of the diversity of the programs that makes me want to see more” (71-year-old man). One participant mentioned their growing interest in jazz, noting, “Jazz is something new and seeing how musicians work with each other and seem to improvise is amazing” (81-year-old woman, In-Person Attendee). This participant went on to note that the unique opportunity provided by LCM to question and learn from performers adds to this appreciation, “Being so close to the musicians makes me appreciate their skill” (81-year-old woman). This is further elucidated in the following quote.

I’m not one much for string quartets. But today’s activity, I felt the performers did a good job explaining their music and how they came about their compositions. So, I found that very interesting...I normally think of classical music, string quartets, classical music as being very staggered. It’s put in print, and this is how you play it. Where their music, and how they do it is very improvisational. And talked about how some parts of their music can change as late as the moment they’re walking out on stage, they’ll come up with an idea and change a piece of what they’re gonna play right on the spot. (71-year-old man, Virtual Attendee).

Another participant described their deep immersion in the music in the following quote.

I don’t even remember the room being with a lot of people... all I feel is I was captured by the musicians. And I was looking at each musician, each instrument and how they were coordinated. I wasn’t looking at the people here around me. It could have been sparse like this...Were there a lot of people there? I don’t know” (70-year-old woman, In-Person Attendee).

This final reflection highlights the program’s ability to transport participants into the world of art, allowing them to lose themselves in the experience and focus on the music and the emotions it evokes. This speaks to the depth of the artistic stimulation pillar beyond learning, appreciating, and enjoying performances, music, and art.

Taking both the qualitative and quantitative results related to artistic stimulation together, we are able to see different perspectives on participant experiences. Care-partner-reported arts engagement for PLWD increased across the season, with exploratory analyses showing an increase for in-person participants but not virtual participants. In contrast, all interviewed PLWD regardless of format described learning, exploring, or appreciating the arts during individual programs. The quantitative measure assessed care partners’ perceptions of PLWD arts engagement over time, whereas the open-ended questions asked PLWD directly about their experiences immediately following a program. Together, these findings suggest that PLWD in both formats experienced artistic stimulation during LCM, although a pre-post increase in care-partner-reported PLWD arts engagement was observed only in the in-person subgroup.

## Discussion

This exploratory mixed-methods program evaluation examined participant experiences with LCM across in-person and virtual formats. Participants in both modalities reported high levels of satisfaction and described experiences consistent with the four LCM pillars of Validation/Personhood, Social Engagement, Self-Expression, and Artistic Stimulation. The findings are preliminary and should be interpreted as participants’ reported experiences and perceived benefits rather than evidence of program effectiveness.

Our findings extend Windle et al., who emphasized the role of trained facilitators, a focus on enjoyment over illness, and accessible, inclusive environments [9]. LCM’s design addresses each of these: professional artists and trained facilitators welcomed and respected all participants, treating them as capable contributors. Qualitative data supported these findings, with participants emphasizing how facilitators made concerted efforts to personally welcome each attendee and recognize their presence, creating an inclusive atmosphere that made everyone feel they belonged. This sense of inclusion was quantitatively supported by comparable social engagement scores across formats, with no significant difference between virtual and in-person modality groups; however, in-person participants reported significantly higher scores on the item “I have friends here” compared to virtual participants. Although overall social engagement scores were similar across formats, the exploratory item-level finding and qualitative responses suggest that participants may have experienced social connection differently across the two formats.

Similar to Malinin et al. [8] and Reel et al. [10], participants described positive experiences of social engagement within LCM, including connections with care partners, other participants, facilitators, and artists. They valued opportunities to connect not only with other attendees but also with their care partners and family members in new and meaningful ways. One virtual participant shared how attending with her daughter deepened their emotional bond, while another described feeling like a source of support for newer attendees. For some, simply being in a space intentionally created for people like them offered a rare and affirming sense of belonging. Even participants who were initially slow to connect described growing more comfortable over time, underscoring the importance of LCM’s ongoing structure.

Opportunities for self-expression are a core component of the LCM program, giving participants the chance to explore their creativity through music, visual arts, and performance. As in Camic et al. [25] and Kontos et al. [26], participants described reconnecting with their creative identities, expressing emotions, and being inspired to resume past creative hobbies. Specifically, participants emphasized how facilitators accepted anything people had to say and created a nonjudgmental environment where PLWD felt free to express themselves artistically without fear of doing it “wrong.” Even those initially hesitant to engage in expressive workshop activities reported greater comfort and willingness to participate over time, again highlighting the value of ongoing programming.

Artistic stimulation was supported in qualitative responses, with participants expressing appreciation for new artistic genres and the opportunity to engage interactively with professional performers. Participants described learning to connect music and visual art as well as how they appreciated being exposed to new forms of music and performing arts. Quantitative findings provided a complementary perspective. Care-partner-reported arts engagement for PLWD increased from pre- to post-season overall, with exploratory analyses identifying an increase among in-person participants but not virtual participants. Because participants were not randomly assigned to modality and the two formats differed in meaningful ways, this pattern should not be interpreted as evidence that in-person programming was more effective than virtual programming. Although artistic stimulation has been mentioned by past researchers, our focus on this concept as both a pillar and evaluative outcome makes this a valuable contribution to the arts-based program literature.

High levels of satisfaction, sustained program participation, and positive qualitative feedback were consistent with program acceptability across both virtual and in-person formats. These preliminary findings are also consistent with prior research highlighting the potential value of dyadic, community-based arts programming [8]. The availability of virtual programming increased accessibility for participants unable to leave the home, as well as those outside of NYC, although opportunities for peer-to-peer connection were somewhat more limited in this modality.

Although virtual participants reported descriptively higher program satisfaction, this difference was not statistically significant. In contrast, some of the exploratory findings related to arts engagement and social connection were stronger for in-person participants. These findings are not necessarily contradictory. Satisfaction, arts engagement, and social connection capture different aspects of the program experience, and the qualitative findings suggest that participants engaged with the two formats in different ways. In-person programming provided more opportunities for interaction with other attendees, while virtual programming provided opportunities for connection from home and shared experiences with care partners and family members. These differences may help explain some of the patterns across outcomes. However, participants selected their modality, and the programs differed in more than delivery format, so we cannot determine whether these differences were due to modality itself.

Our findings also offer support for relational wellbeing as an outcome of arts-based programming for people living with dementia and their care partners. Relational wellbeing emphasizes connection and meaning-making within relationships rather than as individual outcomes [16,17]. Across both in-person and virtual participants, PLWD described experiences that involved shared reflection, joint participation, and mutual enjoyment. Participants described feeling more connected to the other attendees as well as their care partners, referencing how the performance and workshop activities provided opportunities for shared enjoyment and discussion. Although person-centered care remains a central guiding principle of many dementia programs, including the validation pillar of the LCM program, the structure and reported experiences within the program also reflect components consistent with relational approaches to care, where both members of the dyad are positioned as equal participants.

### Implications for arts-based programs

Our findings suggest that multi-modal, dyadic arts interventions such as LCM may provide opportunities for experiences related to personhood, social engagement, self-expression, and artistic stimulation for PLWD and their care partners. Aspects of participants’ experiences may be useful for practitioners and program designers. For example, participants’ descriptions highlighted the value of sustained programming. Some participants who were initially hesitant to engage described becoming more comfortable and willing to participate over time. The ongoing nature of LCM may also help address concerns that can arise when short-term programs end, as noted by previous researchers [15]. Participants’ responses also highlighted the value of including care partners in programming. PLWD described connecting not only with other attendees but also with their care partners and family members through shared participation. For example, participants described attending alongside a care partner, talking together about the experience, and sharing meaningful emotional experiences with family members. These findings are consistent with previous research emphasizing the potential value of shared participation for dementia care dyads [8].

The qualitative findings also point to the importance of how programs are facilitated. Participants frequently described facilitators as welcoming, accepting, and supportive of different levels of participation. In particular, participants valued being able to contribute without fear of giving a wrong answer or being judged. This suggests that facilitator training for arts-based dementia programs should extend beyond artistic expertise to include strategies for supporting autonomy, communication, and participation among PLWD [9]. Participants’ accounts of reconnecting with creative interests and becoming more willing to participate further illustrate the importance of creating an environment in which different forms and levels of engagement are accepted.

Accessibility and modality flexibility are also important considerations. The two formats appeared to offer somewhat different opportunities for social engagement. In-person programming created more opportunities for participants to interact directly with other attendees, develop friendships, exchange ideas, and feel part of a group. Virtual programming appeared to support connection in different ways, including participation alongside family members, shared experiences with care partners, and exposure to a broader community of people they may not otherwise encounter. For practitioners, this suggests that virtual programming should not simply reproduce an in-person format online. Instead, program designers may want to intentionally build in opportunities for peer interaction, introductions, and small-group discussion while preserving the flexibility and accessibility that virtual participation provides. In both formats, participants also emphasized the importance of welcoming staff, multiple ways to participate, and an environment where they felt comfortable contributing. These features reflect Hatton’s [16] emphasis on attending to the environmental and contextual factors that shape relational arts programming for PLWD.

### Limitations

Several limitations should be noted. First, our sample size, although comparable to similar community-based studies, limits the generalizability and transferability of our findings. Additionally, the in-person programming was offered in an urban setting with the benefit of Lincoln Center’s extensive resources, visibility, and partnerships that may be difficult to replicate at the same scale in other settings—particularly in rural areas or less-resourced communities. As noted in Windle et al. [9] and Malinin et al. [8], many arts-based intervention studies face similar challenges related to recruitment and retention. Participants also self-selected into LCM and our study, and we did not include a control or comparison group. Consequently, observed pre-post differences cannot be attributed to participation in LCM, and participants who chose to participate may have differed from other eligible dyads in ways that influenced their experiences and responses. Second, although we captured descriptive qualitative data from PLWD, the quantitative measures were developed specifically for this program evaluation and have not undergone formal psychometric validation. Although internal consistency was acceptable in the current sample, these simplified rating scales may not fully capture nuanced changes across the season. Future researchers should conduct further analyses on the psychometric properties of these measures with an appropriate sample size.

Third, this study focused primarily on an evaluation of the LCM Pillars; future work should include more comprehensive assessments of program outcomes for both the PLWD and their care partner. We did not control for prior arts engagement, caregiver burden, dementia severity, or type of dementia diagnosis, which may have influenced participants’ responsiveness. Participants with different levels of cognitive impairment may differ in how they engage with and respond to arts programming. Without this information, we cannot determine whether participants’ experiences or outcomes differed based on dementia severity. Finally, participants were not randomly assigned to virtual or in-person programming, and the two formats differed in ways beyond delivery modality (e.g., including program content, structure, activities, and opportunities for interaction with performers and other participants). Participants who selected each format may also have differed in characteristics that were not measured in this study. Therefore, any potential differences between modality groups cannot be attributed to delivery format itself, and these comparisons should be considered exploratory. Future researchers should examine how modality-specific program components and participant characteristics may relate to outcomes.
