# Supplementary material for: “It absolutely enlarged my world”: Exploring in-person and virtual performing arts programming for dementia care dyads
Source: PLOS Ment Health. 2026 Nov 3;3(11):e0000771. doi: 10.1371/journal.pmen.0000771 (PMC13730334; doi:10.1371/journal.pmen.0000771)
Supplement: Supplementary file 1 [file pmen.0000771.s002.docx]

Dear Editor and Reviewers,

We sincerely thank the editor and both reviewers for taking the time to read our manuscript and provide detailed, constructive feedback. We appreciate the reviewers’ recognition of the importance of this work and their thoughtful suggestions for strengthening the manuscript. We have revised the manuscript substantially in response to these comments. In particular, we expanded the description of the Lincoln Center Moments program and study procedures, clarified recruitment and measurement, strengthened reporting of the quantitative and qualitative analyses, reframed the study as an exploratory mixed-methods program evaluation, and revised the Discussion and Implications to avoid causal or effectiveness claims. We also addressed data availability, terminology, formatting, and presentation throughout the manuscript.

Below, we respond point by point to the journal requirements and reviewer comments. We are grateful for the time and care invested in the review process and believe these revisions have substantially strengthened the manuscript.

Journal Requirements:

1. Your manuscript is missing the following sections: Introduction. Please ensure these are present, and in the correct order, and that any references to subheadings in your main text are correct. An outline of the required sections can be consulted in our submission guidelines here: [LINK]

<https://journals.plos.org/mentalhealth/s/submission-guidelines#loc-parts-of-a-submission>

- The introductory material was included in the original manuscript but was inadvertently missing the required “Introduction” section heading. We have added the heading “Introduction” before the opening paragraph of the main text. The existing introductory subsections (Dyadic and Participatory Arts Programs, The Lincoln Center Moments Program, Theoretical Framework, and The Current Study) now appear under this heading. No substantive content was added in response to this formatting correction.

2. Thank you for uploading your study's underlying data set. Unfortunately, the repository you have noted in your Data Availability statement does not qualify as an acceptable data repository according to PLOS's standards.

At this time, please upload the minimal data set necessary to replicate your study's findings to a stable, public repository (such as figshare or Dryad) and provide us with the relevant URLs, DOIs, or accession numbers that may be used to access these data. For a list of recommended repositories and additional information on PLOS standards for data deposition, please see <https://journals.plos.org/plosone/s/recommended-repositories>.

- We reviewed PLOS's recommended repository guidance and confirmed that the Open Science Framework (OSF) is listed as an acceptable cross-disciplinary repository. We have retained OSF as the repository for this study and have taken additional steps to ensure that the deposited materials meet PLOS's data availability requirements. Specifically, we have made the deidentified minimal dataset necessary to reproduce the quantitative findings publicly available and have minted a persistent DOI for the OSF project. The dataset and supporting study materials are available at <https://doi.org/10.17605/OSF.IO/XSTVG>. Full qualitative interview transcripts are not publicly shared because of the potential for participant identification within this small population; however, the qualitative codebook, study materials, and quotations supporting the reported findings are publicly available. A data availability statement has been added to the manuscript.

**Reviewers' Comments**

Reviewer's Responses to Questions

**Comments to the Author**

1. Does this manuscript meet PLOS Mental Health’s [publication criteria](https://track.editorialmanager.com/CL0/https:%2F%2Fjournals.plos.org%2Fmentalhealth%2Fs%2Fcriteria-for-publication/1/010f019f601511fa-5bef4bcc-8d15-4c10-ba7e-4c5e66824d4f-000000/HMBg5A9V5fCIL68X3l8LtMP4w8QyGtY08TWsnEW4Q2E=258)? Is the manuscript technically sound, and do the data support the conclusions? The manuscript must describe methodologically and ethically rigorous research with conclusions that are appropriately drawn based on the data presented.

Reviewer #1: Partly

Reviewer #2: Partly

2. Has the statistical analysis been performed appropriately and rigorously?

Reviewer #1: I don't know

Reviewer #2: No

3. Have the authors made all data underlying the findings in their manuscript fully available (please refer to the Data Availability Statement at the start of the manuscript PDF file)?

The [PLOS Data policy](https://track.editorialmanager.com/CL0/https:%2F%2Fjournals.plos.org%2Fmentalhealth%2Fs%2Fdata-availability/1/010f019f601511fa-5bef4bcc-8d15-4c10-ba7e-4c5e66824d4f-000000/uMZf6nhvxKFcUltDUNcmiYjvLRZ6lYswwDkXJmwW-6k=258) requires authors to make all data underlying the findings described in their manuscript fully available without restriction, with rare exception. The data should be provided as part of the manuscript or its supporting information, or deposited to a public repository. For example, in addition to summary statistics, the data points behind means, medians and variance measures should be available. If there are restrictions on publicly sharing data—e.g. participant privacy or use of data from a third party—those must be specified.

Reviewer #1: Yes

Reviewer #2: No

4. Is the manuscript presented in an intelligible fashion and written in standard English?

PLOS Mental Health does not copyedit accepted manuscripts, so the language in submitted articles must be clear, correct, and unambiguous. Any typographical or grammatical errors should be corrected at revision, so please note any specific errors here.

Reviewer #1: Yes

Reviewer #2: Yes

5. Review Comments to the Author

Please use the space provided to explain your answers to the questions above. You may also include additional comments for the author, including concerns about dual publication, research ethics, or publication ethics. (Please upload your review as an attachment if it exceeds 20,000 characters)

Reviewer #1: The study addresses an interesting and clinically relevant non-pharmacological intervention. A particular strength is that the intervention is described as a structured programme, which appears potentially replicable in similar clinical or care settings. Precisely because of this potential applicability, however, the manuscript would benefit from greater clarity and methodological detail regarding both the intervention itself and the outcome measures and analyses performed.

Specifically, the authors should provide a more detailed description of the intervention components, including their rationale, setting, frequency, duration, mode of delivery, professional roles involved, and any procedures used to ensure fidelity or consistency across participants. In addition, the manuscript should more clearly specify the measures used to assess outcomes, the timing of assessments, and the analyses conducted (quantitative and qualitative). Greater transparency in these areas would improve the reader’s ability to evaluate the robustness of the findings and the extent to which the intervention could be reproduced in comparable settings.

- We expanded the Introduction and Methods section to provide more detail about both the LCM program and the study procedures. We now describe the four guiding pillars of LCM, program setting, frequency and duration, virtual and in-person modes of delivery, examples of programming, the structure of performances and interactive workshops, and the professional backgrounds and roles of program facilitators. We also provide additional information about facilitator preparation and procedures used to promote consistency and accessibility across programs.
- We also expanded the Procedure and Measures sections to specify who completed each assessment and when it was administered. Care partners completed baseline and post-season assessments of their own arts engagement and proxy assessments of PLWD arts engagement, whereas PLWD completed brief quantitative ratings of program satisfaction and social engagement and open-ended interviews immediately following individual programs. We provide additional information about measure development, scoring, sample items, and internal consistency.
- Finally, we expanded the Data Analysis section to provide greater transparency regarding both quantitative and qualitative analyses. We now identify the statistical software used, distinguish planned pre-post analyses from exploratory modality comparisons, describe calculation of composite scores, handling of missing data, and report analytic sample sizes. For the qualitative analysis, we describe the evaluative coding approach, number of coders, collaborative development and refinement of the codebook, consensus procedures used to promote coding consistency, and the incorporation of inductively identified patterns. These revisions were made to provide sufficient methodological detail for readers to evaluate the study and understand how the program and evaluation procedures could be reproduced.

Authors should also check redundancies and coherence of the terms used in the text. For instance, the abbreviation "PWD" is sometimes replaced by "PLWD".

- Thank you for noting this. We reviewed the manuscript for redundant text, corrected remaining typographical and formatting inconsistencies, and standardized terminology throughout. We now use PLWD consistently when referring to people/persons living with dementia.

Reviewer #2: This is important research and highlights an underrepresented population. Thank you for submitting. Great job adapting research practices to dementia-impacted individuals and focusing on accessibility in both the program and the research process. The manuscript would be stronger with clearer framing as an exploratory mixed-methods program evaluation avoiding language around “evidence of program effectiveness”. Given the small sample size, self-selection into the program, lack of randomization, and no control/comparison group, the findings should be described as preliminary, exploratory, and based on self-report/perceived benefit metrics, with the implied implications. I believe the manuscript requires major revision in different areas. See below comments and revisions by section:

- We revised the manuscript throughout to more clearly frame the study as an exploratory mixed-methods program evaluation rather than an evaluation of program effectiveness. Specifically, we revised the Abstract, Introduction, Methods, Discussion, Implications, and Limitations to remove or qualify language that could imply that participation in LCM caused the observed outcomes. We now consistently describe the quantitative findings as preliminary and exploratory and interpret them alongside participants’ reported experiences and care-partner proxy reports rather than as evidence of program effectiveness.
- We also strengthened discussion of the methodological limitations that inform this interpretation. The revised manuscript acknowledges the small sample size, self-selection into the program and research study, absence of a control or comparison group, and lack of random assignment to virtual or in-person programming. We further clarify that the virtual and in-person programs differed in ways beyond delivery modality and that participants selecting each format may have differed on unmeasured characteristics. Accordingly, differences between modality groups are described as exploratory and are not attributed to the delivery format itself.
- Finally, we revised the Discussion and Implications to focus more closely on what participants reported experiencing and on potential considerations for future arts-based programming. For example, rather than interpreting modality differences as evidence that one format was more effective, we discuss the different opportunities for social engagement described by participants across formats and their potential implications for program design. In-person participants more often described interactions with other attendees, friendship development, and becoming part of a group, whereas virtual participants described connection through care partners and family members and access to a broader community. We believe these revisions better align the interpretation and implications of the findings with the exploratory nature of the study.

Intro:
* In the introduction, please add a brief description of dementia and the impact of this condition at the psychosocial level. This should answer the “why” this population, anchored in identifying a specific need.
* Please include the impact of dementia not only on the person living with dementia, but also on care partners and dyadic relationships.

- Thank you for these suggestions. We added a brief description of dementia and its impact on psychosocial wellbeing to the beginning of the Introduction. We also added discussion of how dementia can affect care partners and the relationship between members of the care dyad. This additional context helps explain the need for programs that support meaningful activity and social connection for both people living with dementia and their care partners.

* Please briefly describe or conceptualize each of the four LCM pillars from the onset: Validation/Personhood, Social Engagement, Self-Expression, and Artistic Stimulation, which would help tie up the narrative when the results are introduced.

- We have expanded the initial description of the LCM program to briefly define each of its four core pillars: Validation/Personhood, Social Engagement, Self-Expression, and Artistic Stimulation. These definitions provide additional context for the program structure and clarify the framework used to organize the results.

Recruitment:
* If available, please explain why from 48 eligible dyads only 23 enrolled. From an implementation science perspective, this is important.
* Please include more detail on recruitment, barriers to enrollment, and reasons for non-participation if these were tracked.
* A flow chart would be helpful across individuals who expressed interest, eligible dyads, contacted dyads, enrolled dyads, participants who attended, participants who completed baseline/follow-up, and participants included in each analysis.

- We revised the Participants section to provide greater transparency regarding recruitment, enrollment, and retention. Importantly, we clarified an ambiguity in the original manuscript regarding the 48 eligible dyads and the final sample of 23 dyads. The 48 eligible dyads were identified during the more systematically tracked Spring 2024 recruitment period, whereas the final sample of 23 dyads included participants from both the Fall 2023 pilot (6 dyads) and Spring 2024 (17 dyads). Thus, the reduction from 48 eligible dyads to 23 participants did not represent a single recruitment funnel across the two seasons.
- For Spring 2024, 72 dyads initially expressed interest through the LCM registration form, of whom 48 met eligibility criteria and were contacted. We have added details regarding the recruitment process, including repeated email and telephone outreach and three information sessions. We also clarify that reasons for non-participation among eligible dyads were not systematically collected and therefore cannot be reported retrospectively. Two additional Spring dyads completed baseline assessments but did not subsequently attend LCM programming or complete follow-up assessments and were not included in the final sample. We have added Figure 1, Participant Recruitment and Study Flow Across Fall 2023 and Spring 2024, to visually summarize recruitment, eligibility, participation, and retention across the two seasons. Because sample sizes differed across quantitative analyses due to missing data, we also report the analytic N for each statistical test in the Results rather than implying that all 23 dyads contributed data to every analysis.

* The manuscript states that dementia type and severity were not assessed. How do you think not assessing for dementia severity impacted the findings of the study?

- Because dementia type and severity were not assessed, we cannot determine whether participants' experiences or perceived benefits differed according to cognitive impairment severity. The sample may have included individuals with heterogeneous levels and types of cognitive impairment, which limits our ability to determine whether the findings generalize across stages or types of dementia. We expanded on how this limits our findings and recommend that future evaluations assess dementia type and severity when feasible while continuing to minimize participant burden.

* Given that clinical documentation was not required, please consider whether the manuscript should consistently refer to participants as people living with dementia or memory loss, rather than only people living with dementia?

- Finally, we considered the reviewer's thoughtful point regarding terminology. LCM is a program designed and marketed for people living with dementia and their care partners, and participants self-identified or were identified by a care partner as living with memory loss; clinical documentation of a dementia diagnosis was not required for study participation. We have clarified this eligibility criterion in the Participants section. We retained the term “people living with dementia” (PLWD) when discussing the program's intended population and the broader dementia literature, while making clear that dementia diagnoses were not independently verified in the current sample. We believe this approach accurately reflects the program context while avoiding the implication that the research team clinically established a dementia diagnosis for each participant.

Methods:
* Kindly specify the number of coders, and clarify whether inter-rater reliability was evaluated and reached. If IRR was not calculated, please explain how coding agreement or consistency was established. Please specify the software used for qualitative analysis.

- Two researchers with experience in qualitative research conducted the coding. Formal inter-rater reliability was not calculated because our analytic process used collaborative, consensus-based coding rather than independent coding designed to estimate statistical coder agreement. The two coders collaboratively coded the Fall 2023 data to develop and refine the codebook and establish a shared understanding of how the evaluative criteria should be applied. Coding decisions and areas of uncertainty were discussed and refined collaboratively throughout the analytic process to promote consistency across coders. We also added information about the transparency of the coding process. The complete codebook is publicly available through our OSF repository and includes participant-level quotations associated with each code, allowing readers to examine the textual evidence underlying the coding decisions. No qualitative analysis software was used; Microsoft Excel was used to organize codes by pillar and connect codes with supporting participant quotations.

* Further, kindly describe data handling procedures

- We have expanded the Procedure section to describe our data handling procedures. The Lincoln Center staff member hired to manage study activities conducted and transcribed the interviews and removed identifying information before providing the qualitative data to the broader research team for analysis. Survey data were collected electronically, and identifying information was similarly removed by Lincoln Center staff before the data were provided to the research team. We also clarify that the researchers responsible for measure development and data analysis were independent of Lincoln Center and were not involved in LCM program development or implementation. The deidentified quantitative data used in the analyses are publicly available through our OSF repository.

* Moreover, I would appreciate if you could clarify who conducted the interviews and whether they were independent from the LCM program.

- We have expanded the Procedure section to specify who conducted the interviews and their relationship to LCM programming. Interviews were conducted by a Lincoln Center staff member hired specifically to oversee research activities for this study, including recruitment, interviewing, transcription, and participant follow-up. Importantly, this individual was not involved in the development, facilitation, or implementation of LCM programming. We have added this information to clarify the separation between program delivery and data collection.

* Kindly clarify whether care partners or staff were present during interviews with participants living with dementia, as well as who completed each measure and from whose perspective. For example, the manuscript states that arts engagement was assessed by care partners, but the results appear to report arts engagement for both people living with dementia and care partners.

- We have revised the Procedure and Measures sections to make the interview context and respondent perspective more explicit. PLWD completed brief interviews immediately following each program they attended. The research interviewer was present with the PLWD during these interviews, while care partners were not included in the post-program interviews. PLWD directly answered the open-ended interview questions as well as the post-program quantitative items assessing program satisfaction and social engagement.
- Care partners completed the demographic questionnaire and longitudinal baseline and final surveys. These surveys included items assessing care partners’ own experiences as well as items asking them to report their perceptions of the PLWD’s engagement and well-being. We now explicitly state that responses provided by care partners about the PLWD represent the care partner’s perspective rather than direct self-report by the PLWD. We have also reviewed the Measures and Results sections to clarify the respondent and perspective associated with each outcome.

* Additional detail about scale construction, number of items, scoring, reliability, etc., would also strengthen the paper. If no use of validated scales was employed, kindly justify this choice, and provide further details about the item generation and selection for the proposed surveys used.

- We expanded the Measures section to provide additional information about measure development, construction, scoring, and reliability. The quantitative measures were developed specifically for this program evaluation to assess participants’ experiences in relation to LCM’s four program pillars. Item development was informed by focus groups conducted in Summer 2023 with PLWD, care partners, and LCM program facilitators. Feedback from these groups was used to identify experiences and outcomes relevant to the program’s intended goals and to develop items that were brief and accessible for the study population.
- Because the primary purpose of the study was to evaluate whether LCM was meeting its specific program goals, study-specific measures were selected rather than broader standardized measures that did not directly map onto the four LCM pillars. We now explain this rationale explicitly. For each quantitative measure, we provide the number of items, response scale, sample items, respondent, timing of administration, scoring procedure, and internal consistency in the current sample. Cronbach’s alpha was .78 for Program Satisfaction, .86 for Engagement with the Arts, and .73 for Social Engagement. The complete study measures and interview guides are also available through our OSF repository. We also broadened our limitations section to note this concern.

Results and interpretation:
* Please clarify:
* Software used for analyses
* the degrees of freedom. There appear to be conflicting numbers across the results.
* analytic N for each test.
* how missing data were handled.

- We have revised the Data Analysis and Results sections and Table 2 to provide additional detail regarding the quantitative analyses. Quantitative analyses were conducted in JASP. We now report the analytic sample size and degrees of freedom for each statistical test. Differences in degrees of freedom reflect differences in the number of participants with data available for each analysis rather than conflicting analyses. For example, the in-person pre-post analyses included 13 participants with complete paired data (*df* = 12), whereas the virtual pre-post analyses included seven participants with complete paired data (*df* = 6). The primary independent-samples comparisons of in-person and virtual participants included 14 in-person and seven virtual participants (*N* = 21; *df* = 19).
- Missing values were not imputed. Analyses were conducted using available observed data, with participants included in an analysis when they had the data required for that specific analysis. For paired pre-post analyses, this required data at both assessment points. Consequently, analytic sample sizes varied across analyses. We have revised Table 2 and the Results to make the analytic sample size associated with each test explicit.

* how analyses were decided, especially given the small sample size and ordinal 4-point response scales.

- Pre-post comparisons of the quantitative outcomes assessed longitudinally were planned as part of the program evaluation. However, comparisons between in-person and virtual participants were exploratory. We have revised the Data Analysis section to make this distinction clearer and to characterize the quantitative analyses as exploratory given the small sample size.
- Although individual responses were collected using 4-point ordinal response scales, the primary quantitative measures were multi-item measures scored by averaging responses across items to create composite scores. Nevertheless, given the small analytic samples and bounded response scales, we conducted nonparametric sensitivity analyses. Wilcoxon signed-rank tests were used for the planned paired-samples comparisons. The increase in care-partner-reported arts engagement among PLWD participating in person was also observed using the Wilcoxon signed-rank test (p = .022), whereas the remaining pre-post comparisons were not statistically significant using either approach. We also conducted Mann-Whitney U tests for the exploratory between-group comparisons. The difference on the “I have friends here” item was also observed using the Mann-Whitney U test (U = 81.50, p = .011), whereas the remaining between-group comparisons were not statistically significant. We revised the manuscript to emphasize effect sizes and confidence intervals and to interpret the quantitative findings as exploratory.

* Kindly include confidence intervals for mean differences and effect sizes where possible

- We have revised Table 2 to report 95% confidence intervals for both mean differences and Cohen’s d effect sizes for the primary quantitative analyses. We also report analytic sample sizes and degrees of freedom in the table to provide a more complete representation of the estimates and their precision.

* Please revise language so the results do not imply causality or effectiveness. These findings are better described as exploratory and preliminary. Please add a section where the quantitative and qualitative results inform each other and tell a coherent story. This is critical in mixed-methods research.

- We revised the manuscript to describe quantitative findings as exploratory and preliminary, and to remove or qualify language that could imply causality or program effectiveness. We revised interpretations of pre-post changes and differences between virtual and in-person participants to avoid suggesting that participation caused observed changes or that one modality was more effective than the other. We also emphasize the small sample size when interpreting these findings.
- We also strengthened the integration of the quantitative and qualitative findings. Because the Results are organized around the four LCM program pillars, we integrated the findings within the relevant pillar sections rather than adding a separate mixed-methods section. For Social Engagement, we consider the similar overall quantitative social engagement scores alongside the item-level and qualitative findings, which suggest that social connection was present in both formats but may have taken different forms. For Artistic Stimulation, we consider the longitudinal care-partner reports alongside PLWD’s direct descriptions of their experiences immediately following individual programs. This integration highlights that the two methods assessed different aspects of participants’ experiences and provides context for areas where the findings converge or differ. Validation/Personhood and Self-Expression were assessed qualitatively and therefore do not include corresponding quantitative findings.

* Kindly take a look at the virtual versus in-person comparisons and describe more clearly:
* Participants were not randomized to modality + formats differed in content, materials, performer interaction, and likely participant characteristics etc. Therefore, the paper should not imply that modality itself caused differences in outcomes.

- We have revised the manuscript to clarify the exploratory nature of the virtual versus in-person comparisons. Participants were not randomly assigned to modality, and the virtual and in-person programs differed in several ways beyond delivery format, including program content, structure, activities, and opportunities for interaction. Participants who selected the two formats may also have differed on characteristics that were not measured. We now state explicitly in the Data Analysis and Limitations sections that differences between groups cannot be attributed to modality itself.

* Virtual and in-person programs seemed to differ in preference (e.g. virtual?), but the results seem to conflict (e.g. better effect sizes for in person), if correct, please describe why this might be happening in the discussion

- We revised the Discussion to address the different patterns observed across outcomes. Although virtual participants reported descriptively higher program satisfaction, this difference was not statistically significant. In contrast, some exploratory findings related to arts engagement and social connection were stronger for in-person participants. We clarify that these findings are not necessarily contradictory because satisfaction, arts engagement, and social connection capture different aspects of the program experience. We also draw on the qualitative findings to provide context for these patterns, including differences in the opportunities for interaction and connection available across formats. Finally, we emphasize that participants selected their modality and that the virtual and in-person programs differed in more than delivery format. Therefore, these findings should not be interpreted as evidence that one modality was preferred or more effective than the other.

Data avail.:
The OSF materials and codebook are helpful, thanks! Please clarify whether the de-identified quantitative data are available and if the data cannot be shared due to privacy or IRB restrictions, please state clearly.

- The deidentified quantitative dataset necessary to reproduce the reported analyses is publicly available through our OSF repository at https://doi.org/10.17605/OSF.IO/XSTVG. The study measures, interview guides, and qualitative codebook are also publicly available. Full qualitative interview transcripts are not publicly shared because they contain potentially identifiable information from participants living with dementia; however, relevant qualitative excerpts supporting the findings are reported in the manuscript and are included in the OSF codebook. We added a Data Availability Statement to the revised manuscript to make these details explicit.

Writing & other details:
Please revise minor typo-related issues, use consistent terminology across the manuscript, (e.g., PWD vs PLWD), and make sure the title is consistent across the submission and manuscript. I would suggest a shorter title, but this is the authors’ choice.

- Thank you. We carefully reviewed the manuscript and corrected typographical and formatting errors throughout. We also standardized terminology, including consistently using PLWD (people/person living with dementia) rather than PWD. We appreciated the reviewer’s suggestion to shorten the title and revised it from “It Absolutely Enlarged My World”: Exploring the Impact of In-Person and Virtual Performing Arts Programming for Dementia Care Dyads to “It Absolutely Enlarged My World”: Exploring In-Person and Virtual Performing Arts Programming for Dementia Care Dyads. This revision also removes the term “impact” to better reflect the exploratory nature of the study.

Congrats! This is a great initiative and a good paper with an important contribution, especially in its qualitative findings. I really appreciated the quotes and centering participant perspectives. However, the manuscript should be revised, particularly to emphasize acceptability, perceived benefits, feasibility, preliminary exploratory findings etc rather than effectiveness. I think after revisions are addressed, this could be a great contribution to the literature on arts-based programs and mental health, with an interesting innovation focusing on dyads.

- Thank you so much for these encouraging comments and for recognizing the value of centering participant perspectives! We appreciate your thoughtful advice and suggestions. In response, we revised the manuscript throughout to emphasize acceptability, perceived experiences and benefits, and preliminary exploratory findings rather than effectiveness.

6. PLOS authors have the option to publish the peer review history of their article ([what does this mean?](https://track.editorialmanager.com/CL0/https:%2F%2Fjournals.plos.org%2Fmentalthealth%2Fs%2Feditorial-and-peer-review-process%23loc-peer-review-history/1/010f019f601511fa-5bef4bcc-8d15-4c10-ba7e-4c5e66824d4f-000000/ICv9c8M8Kh490bJJcmSKGcuJhDdF10QfyEwEJlLqhrQ=258)). If published, this will include your full peer review and any attached files.


**Do you want your identity to be public for this peer review?** If you choose “no”, your identity will remain anonymous but your review may still be made public.


For information about this choice, including consent withdrawal, please see our [Privacy Policy](https://track.editorialmanager.com/CL0/https:%2F%2Fwww.plos.org%2Fprivacy-policy/1/010f019f601511fa-5bef4bcc-8d15-4c10-ba7e-4c5e66824d4f-000000/72M3JIvi4Wyfd7KZlxX48gW7-Cb1aY39H1SLfCLgUaA=258).

Reviewer #1: **Yes:** Anna Messina

Reviewer #2: No
