# Supplementary material for: “It absolutely enlarged my world”: Exploring in-person and virtual performing arts programming for dementia care dyads
Source: PLOS Ment Health. 2026 Nov 3;3(11):e0000771. doi: 10.1371/journal.pmen.0000771 (PMC13730334; doi:10.1371/journal.pmen.0000771)
Supplement: Peer review history

# Peer review: Decision Letter 0

**DOI:** 10.1371/journal.pmen.0000771.r001

**Authors:** Ariel Teles

14 Jul 2026

PMEN-D-26-00054

“It Absolutely Enlarged My World”: Exploring the Impact of In-Person and Virtual Performing Arts Programming for Dementia Care Dyads

PLOS Mental Health

Dear Dr. Walker,

Thank you for submitting your manuscript to PLOS Mental Health. After careful consideration, we feel that it has merit but does not fully meet PLOS Mental Health’s publication criteria as it currently stands. Therefore, we invite you to submit a revised version of the manuscript that addresses the points raised during the review process.

The manuscript was well received. Consider the reviewers' comments.

Please submit your revised manuscript by Sep 12 2026 11:59PM. If you will need more time than this to complete your revisions, please reply to this message or contact the journal office at mentalhealth@plos.org. When you're ready to submit your revision, log on to https://www.editorialmanager.com/pmen/ and select the 'Submissions Needing Revision' folder to locate your manuscript file.

Please include the following items when submitting your revised manuscript:

- A letter that responds to each point raised by the editor and reviewer(s). You should upload this letter as a separate file labeled 'Response to Reviewers'.
- A marked-up copy of your manuscript that highlights changes made to the original version. You should upload this as a separate file labeled 'Revised Manuscript with Track Changes'.
- An unmarked version of your revised paper without tracked changes. You should upload this as a separate file labeled 'Manuscript'.

Guidelines for resubmitting your figure files are available below the reviewer comments at the end of this letter.

As the corresponding author, your ORCID iD is verified in the submission system and will appear in the published article. PLOS supports the use of ORCID, and we encourage all coauthors to register for an ORCID iD and use it as well. Please encourage your coauthors to verify their ORCID iD within the submission system before final acceptance, as unverified ORCID iDs will not appear in the published article. *Only*  the individual author can complete the verification step; PLOS staff *cannot*  verify ORCID iDs on behalf of authors.

We look forward to receiving your revised manuscript.

Kind regards,

Ariel Soares Teles

Academic Editor

PLOS Mental Health

Journal Requirements:

1. Your manuscript is missing the following sections: Introduction. Please ensure these are present, and in the correct order, and that any references to subheadings in your main text are correct. An outline of the required sections can be consulted in our submission guidelines here: [LINK]

https://journals.plos.org/mentalhealth/s/submission-guidelines#loc-parts-of-a-submission

2. Thank you for uploading your study's underlying data set. Unfortunately, the repository you have noted in your Data Availability statement does not qualify as an acceptable data repository according to PLOS's standards.

At this time, please upload the minimal data set necessary to replicate your study's findings to a stable, public repository (such as figshare or Dryad) and provide us with the relevant URLs, DOIs, or accession numbers that may be used to access these data. For a list of recommended repositories and additional information on PLOS standards for data deposition, please see https://journals.plos.org/plosone/s/recommended-repositories.

If the reviewer comments include a recommendation to cite specific previously published works, please review and evaluate these publications to determine whether they are relevant and should be cited. There is no requirement to cite these works unless the editor has indicated otherwise.

Additional Editor Comments (if provided):

[Note: HTML markup is below. Please do not edit.]

Reviewers' comments:

Reviewer's Responses to Questions

**Comments to the Author**

1. Does this manuscript meet PLOS Mental Health’s publication criteria? Is the manuscript technically sound, and do the data support the conclusions? The manuscript must describe methodologically and ethically rigorous research with conclusions that are appropriately drawn based on the data presented.

Reviewer #1: Partly

Reviewer #2: Partly

**********

2. Has the statistical analysis been performed appropriately and rigorously?

Reviewer #1: I don't know

Reviewer #2: No

**********

3. Have the authors made all data underlying the findings in their manuscript fully available (please refer to the Data Availability Statement at the start of the manuscript PDF file)?

The PLOS Data policy requires authors to make all data underlying the findings described in their manuscript fully available without restriction, with rare exception. The data should be provided as part of the manuscript or its supporting information, or deposited to a public repository. For example, in addition to summary statistics, the data points behind means, medians and variance measures should be available. If there are restrictions on publicly sharing data—e.g. participant privacy or use of data from a third party—those must be specified.

Reviewer #1: Yes

Reviewer #2: No

**********

4. Is the manuscript presented in an intelligible fashion and written in standard English?

PLOS Mental Health does not copyedit accepted manuscripts, so the language in submitted articles must be clear, correct, and unambiguous. Any typographical or grammatical errors should be corrected at revision, so please note any specific errors here.

Reviewer #1: Yes

Reviewer #2: Yes

**********

5. Review Comments to the Author

Please use the space provided to explain your answers to the questions above. You may also include additional comments for the author, including concerns about dual publication, research ethics, or publication ethics. (Please upload your review as an attachment if it exceeds 20,000 characters)

Reviewer #1: The study addresses an interesting and clinically relevant non-pharmacological intervention. A particular strength is that the intervention is described as a structured programme, which appears potentially replicable in similar clinical or care settings. Precisely because of this potential applicability, however, the manuscript would benefit from greater clarity and methodological detail regarding both the intervention itself and the outcome measures and analyses performed.

Specifically, the authors should provide a more detailed description of the intervention components, including their rationale, setting, frequency, duration, mode of delivery, professional roles involved, and any procedures used to ensure fidelity or consistency across participants. In addition, the manuscript should more clearly specify the measures used to assess outcomes, the timing of assessments, and the analyses conducted (quantitative and qualitative). Greater transparency in these areas would improve the reader’s ability to evaluate the robustness of the findings and the extent to which the intervention could be reproduced in comparable settings.

Authors should also check redundancies and coherence of the terms used in the text. For instance, the abbreviation "PWD" is sometimes replaced by "PLWD".

Reviewer #2: This is important research and highlights an underrepresented population. Thank you for submitting. Great job adapting research practices to dementia-impacted individuals and focusing on accessibility in both the program and the research process.The manuscript would be stronger with clearer framing as an exploratory mixed-methods program evaluation avoiding language around “evidence of program effectiveness”. Given the small sample size, self-selection into the program, lack of randomization, and no control/comparison group, the findings should be described as preliminary, exploratory, and based on self-report/perceived benefit metrics, with the implied implications. I believe the manuscript requires major revision in different areas. See below comments and revisions by section:

Intro:

* In the introduction, please add a brief description of dementia and the impact of this condition at the psychosocial level. This should answer the “why” this population, anchored in identifying a specific need.

* Please include the impact of dementia not only on the person living with dementia, but also on care partners and dyadic relationships.

* Please briefly describe or conceptualize each of the four LCM pillars from the onset: Validation/Personhood, Social Engagement, Self-Expression, and Artistic Stimulation, which would help tie up the narrative when the results are introduced.

Recruitment:

* If available, please explain why from 48 eligible dyads only 23 enrolled. From an implementation science perspective, this is important.

* Please include more detail on recruitment, barriers to enrollment, and reasons for non-participation if these were tracked.

* A flow chart would be helpful across individuals who expressed interest, eligible dyads, contacted dyads, enrolled dyads, participants who attended, participants who completed baseline/follow-up, and participants included in each analysis.

* The manuscript states that dementia type and severity were not assessed. How do you think not assessing for dementia severity impacted the findings of the study?

* Given that clinical documentation was not required, please consider whether the manuscript should consistently refer to participants as people living with dementia or memory loss, rather than only people living with dementia?

Methods:

* Kindly specify the number of coders, and clarify whether inter-rater reliability was evaluated and reached. If IRR was not calculated, please explain how coding agreement or consistency was established. Please specify the software used for qualitative analysis.

* Further, kindly describe data handling procedures

* Moveorver, I would appreciate if you could clarify who conducted the interviews and whether they were independent from the LCM program.

* Kindly clarify whether care partners or staff were present during interviews with participants living with dementia, as well as who completed each measure and from whose perspective. For example, the manuscript states that arts engagement was assessed by care partners, but the results appear to report arts engagement for both people living with dementia and care partners.

* Additional detail about scale construction, number of items, scoring, reliability, etc., would also strengthen the paper. If no use of validated scales was employed, kindly justify this choice, and provide further details about the item generation and selection for the proposed surveys used.

Results and interpretation:

* Please clarify:

* Software used for analyses

* the degrees of freedom. There appear to be conflicting numbers across the results.

* analytic N for each test.

* how missing data were handled.

* how analyses were decided, especially given the small sample size and ordinal 4-point response scales.

* Kindly include confidence intervals for mean differences and effect sizes where possible

* Please revise language so the results do not imply causality or effectiveness. These findings are better described as exploratory and preliminary. Please add a section where the quantitative and qualitative results inform each other and tell a coherent story. This is critical in mixed-methods research.

* Kindly take a look at the virtual versus in-person comparisons and describe more clearly:

* Participants were not randomized to modality + formats differed in content, materials, performer interaction, and likely participant characteristics etc. Therefore, the paper should not imply that modality itself caused differences in outcomes.

* Virtual and in-person programs seemed to differ in preference (e.g. virtual?), but the results seem to conflict (e.g. better effect sizes for in person), if correct, please describe why this might be happening in the discussion

Data avail.:

The OSF materials and codebook are helpful, thanks! Please clarify whether the de-identified quantitative data are available and if the data cannot be shared due to privacy or IRB restrictions, please state clearly.

Writing & other details:

Please revise minor typo-related issues, use consistent terminology across the manuscript, (e.g., PWD vs PLWD), and make sure the title is consistent across the submission and manuscript. I would suggest a shorter title, but this is the authors’ choice.

Congrats! This is a great initiative and a good paper with an important contribution, especially in its qualitative findings. I really appreciated the quotes and centering participant perspectives. However, the manuscript should be revised, particularly to emphasize acceptability, perceived benefits, feasibility, preliminary exploratory findings etc rather than effectiveness. I think after revisions are addressed, this could be a great contribution to the literature on arts-based programs and mental health, with an interesting innovation focusing on dyads.

**********

6. PLOS authors have the option to publish the peer review history of their article (what does this mean?). If published, this will include your full peer review and any attached files.

**Do you want your identity to be public for this peer review?**  If you choose “no”, your identity will remain anonymous but your review may still be made public.

For information about this choice, including consent withdrawal, please see our Privacy Policy.

Reviewer #1: **Yes:**  Anna Messina

Reviewer #2: No

**********

[NOTE: If reviewer comments were submitted as an attachment file, they will be attached to this email and accessible via the submission site. Please log into your account, locate the manuscript record, and check for the action link "View Attachments". If this link does not appear, there are no attachment files.]

Figure Resubmissions:

While revising your submission, we strongly recommend that you use PLOS’s NAAS tool (https://ngplosjournals.pagemajik.ai/artanalysis) to test your figure files. NAAS can convert your figure files to the TIFF file type and meet basic requirements (such as print size, resolution), or provide you with a report on issues that do not meet our requirements and that NAAS cannot fix.

After uploading your figures to PLOS’s NAAS tool - https://ngplosjournals.pagemajik.ai/artanalysis, NAAS will process the files provided and display the results in the "Uploaded Files" section of the page as the processing is complete. If the uploaded figures meet our requirements (or NAAS is able to fix the files to meet our requirements), the figure will be marked as "fixed" above. If NAAS is unable to fix the files, a red "failed" label will appear above. When NAAS has confirmed that the figure files meet our requirements, please download the file via the download option, and include these NAAS processed figure files when submitting your revised manuscript.

# Author comment: Author response to Decision Letter 1

**DOI:** 10.1371/journal.pmen.0000771.r002

12 Sep 2026

Attachment Submitted filename: Response to Reviewers FINAL.docx

# Peer review: Decision Letter 1

**DOI:** 10.1371/journal.pmen.0000771.r003

**Authors:** Ariel Teles

14 Oct 2026

“It Absolutely Enlarged My World”: Exploring In-Person and Virtual Performing Arts Programming for Dementia Care Dyads

PMEN-D-26-00054R1

Dear Dr Walker,

We are pleased to inform you that your manuscript '“It Absolutely Enlarged My World”: Exploring In-Person and Virtual Performing Arts Programming for Dementia Care Dyads' has been provisionally accepted for publication in PLOS Mental Health.

Before your manuscript can be formally accepted you will need to complete some formatting changes, which you will receive in a follow up email. A member of our team will be in touch with a set of requests.

Please note that your manuscript will not be scheduled for publication until you have made the required changes, so a swift response is appreciated.

IMPORTANT: The editorial review process is now complete. PLOS will only permit corrections to spelling, formatting or significant scientific errors from this point onwards. Requests for major changes, or any which affect the scientific understanding of your work, will cause delays to the publication date of your manuscript.

If your institution or institutions have a press office, please notify them about your upcoming paper to help maximize its impact. If they'll be preparing press materials, please inform our press team as soon as possible -- no later than 48 hours after receiving the formal acceptance. Your manuscript will remain under strict press embargo until 2 pm Eastern Time on the date of publication. For more information, please contact mentalhealth@plos.org.

Thank you again for supporting Open Access publishing; we are looking forward to publishing your work in PLOS Mental Health.

Best regards,

Ariel Soares Teles

Academic Editor

PLOS Mental Health

***********************************************************

Reviewer Comments (if any, and for reference):

Reviewer's Responses to Questions

**Comments to the Author**

1. If the authors have adequately addressed your comments raised in a previous round of review and you feel that this manuscript is now acceptable for publication, you may indicate that here to bypass the “Comments to the Author” section, enter your conflict of interest statement in the “Confidential to Editor” section, and submit your "Accept" recommendation.

Reviewer #1: All comments have been addressed

Reviewer #2: All comments have been addressed

**********

2. Does this manuscript meet PLOS Mental Health’s publication criteria? Is the manuscript technically sound, and do the data support the conclusions? The manuscript must describe methodologically and ethically rigorous research with conclusions that are appropriately drawn based on the data presented.

Reviewer #1: Yes

Reviewer #2: Yes

**********

3. Has the statistical analysis been performed appropriately and rigorously?

Reviewer #1: N/A

Reviewer #2: Yes

**********

4. Have the authors made all data underlying the findings in their manuscript fully available (please refer to the Data Availability Statement at the start of the manuscript PDF file)?

The PLOS Data policy requires authors to make all data underlying the findings described in their manuscript fully available without restriction, with rare exception. The data should be provided as part of the manuscript or its supporting information, or deposited to a public repository. For example, in addition to summary statistics, the data points behind means, medians and variance measures should be available. If there are restrictions on publicly sharing data—e.g. participant privacy or use of data from a third party—those must be specified.

Reviewer #1: Yes

Reviewer #2: (No Response)

**********

5. Is the manuscript presented in an intelligible fashion and written in standard English?

PLOS Mental Health does not copyedit accepted manuscripts, so the language in submitted articles must be clear, correct, and unambiguous. Any typographical or grammatical errors should be corrected at revision, so please note any specific errors here.

Reviewer #1: Yes

Reviewer #2: Yes

**********

6. Review Comments to the Author

Please use the space provided to explain your answers to the questions above. You may also include additional comments for the author, including concerns about dual publication, research ethics, or publication ethics. (Please upload your review as an attachment if it exceeds 20,000 characters)

**Reviewer #1:**  I would like to thank the authors for their efforts in revising and improving the manuscript. The revised version is now better structured, with greater clarity in the methodological description, which enhances the reproducibility of the study. Furthermore, the implications of the findings are now more clearly presented and discussed, strengthening the overall quality and relevance of the manuscript.

**Reviewer #2:**  The authors have responded thoughtfully and substantively to my previous comments, and the revised manuscript is considerably improved. In particular, the manuscript now more clearly frames the study as an exploratory mixed-methods program evaluation, provides substantially greater detail regarding the intervention, recruitment and enrollment procedures, participant assessment, interviewer role, measurement development, qualitative coding procedures, statistical analyses, missing data, and the limitations associated with the small, non-randomized sample. The addition of nonparametric sensitivity analyses, confidence intervals, analytic sample sizes, and clearer integration of the quantitative and qualitative findings also improves the transparency and interpretability of the results. Congrats on a great manuscript!

**********

7. PLOS authors have the option to publish the peer review history of their article (what does this mean?). If published, this will include your full peer review and any attached files.

**Do you want your identity to be public for this peer review?**  If you choose “no”, your identity will remain anonymous but your review may still be made public.

For information about this choice, including consent withdrawal, please see our Privacy Policy.

Reviewer #1: No

Reviewer #2: No

**********
